# Spermatorrhea

**Published:** 1853-10

**Authors:** 


*Spermatorrhea.* By Professor DUGAS.—There are doubtless but few of our readers who will not feel interested in the articles contained in this number, on the subject of spermatorrhea, for we know of no cases with which we are so continually and painfully annoyed as those in which the patients complain of the real or the imaginary forms of this affection. Indeed, the mind is so uniformly disturbed in such cases that it is often impossible to determine the true state of things, and therefore difficult to know whether to administer relief to the brain or to the generative apparatus. We are so well satisfied, however, that many of the cases brought to our notice have been seriously injured by the injudicious use of Lallemand's favorite remedy—cauterization—that we feel it a duty to raise our voice against such treatment, except as a last resort. Our objection to the use of the nitrate of silver is, that it does not unfrequently occasion strictures and inflammation about the neck of the bladder —either of which may prove to be more serious than the original affection. Such results would, of course, not often follow the application, if made by a skillful and experienced operator, but in the hands of the rash it cannot but be extremely hazardous.

We are free to confess that we have no more confidence in the efficacy of digitaline than in that of other narcotics, as opium, belladonna, tobacco, etc., for they are all antaphrodisiacs to a certain degree. The suggestion of the mechanical means indicated in one of the articles alluded to, seems to us a good one; but care should be taken not to expose the organ to strangulation, in the event of an erection, without sufficient warning to the patient. The best prescription we know of, is one which, in our land of plenty, can fortunately be almost always carried out without inconvenience, and one which we have never known to fail in establishing a radical cure. We mean matrimony. The only difficulty is to convince the patients that they may form this connexion *before* they get cured.

We have found much advantage from the cold hip-bath night and morning, and the injection two or three times a day of a solution of sulphate of morphia into the urethra. The solution may contain 4 grains in 8 ounces of water, a small syringe full of which should be thrown in and retained, by closing the orifice, at least ten minutes, so that it may run up to, or into, the bladder, and remain long enough to produce its sedative effect. The tonic action of the cold bath and the locally sedative effect of the morphia, will tend to lessen the irritability of the organs upon which depends, in many instances, the involuntary emissions. If the proportions suggested affect the system materially, the solution should be made milder.—*Southern Med. and Sur. Journal.*

